# Factors associated with upper extremity use after stroke: a scoping review of accelerometry studies

**DOI:** 10.1186/s12984-025-01568-1

**Published:** 2025-02-24

**Authors:** Léandre Gagné-Pelletier, Isabelle Poitras, Marc Roig, Catherine Mercier

**Affiliations:** 1 School of Rehabilitation Sciences, Université Laval, Quebec City, QC G1V 0A6 Canada; 2 Centre for Interdisciplinary Research in Rehabilitation and Social Integration (Cirris), Centre Intégré Universitaire de Santé et Services Sociaux de la Capitale-Nationale, 525 boul. Hamel, Québec City, QC G1M 2S8 Canada; 3 School of Physical & Occupational Therapy, McGill University, Montreal, Qc H3G 1Y5 Canada; 4 Memory and Motor Rehabilitation Laboratory (Memory-Lab), Center for Interdisciplinary Research in Rehabilitation of Greater Montreal (CRIR), Montreal, Qc H3S 1M9 Canada

**Keywords:** Rehabilitation, Upper limb, Cerebrovascular accident, Accelerometry, Sensors, Use ratio

## Abstract

**Background:**

A discrepancy between the level of impairment at the upper extremity (UE) and its use in activities of daily life is frequently observed in individuals who have experienced a stroke. Wrist-worn accelerometers allow an objective and valid measure of UE use in everyday life. Accelerometer studies have shown that a wide range of factors beyond UE impairment can influence UE use. This scoping review aims to identify factors associated with UE use and to investigate the influence of different types of accelerometry metrics on these associations.

**Method:**

A search using CINHAL, Embase, MEDLINE, Compendex, and Web of Science Core Collection databases was performed. Studies that assessed the association between UE use quantified with accelerometers and factors related to the person or their environment in individuals with stroke were included. Data related to study design, participants characteristics, accelerometry methodology (absolute vs. relative UE use metrics), and associations with personal and environmental factors were extracted.

**Results:**

Fifty-four studies were included. Multiple studies consistently reported associations between relative UE use and stroke severity, UE motor impairment, unimanual capacity, bimanual capacity, and mobility. In contrast, there were inconsistent associations with factors such as neglect and concordance between dominance and side of paresis and a consistent lack of association between relative UE use and time since stroke, sex, and age. Metrics of absolute paretic UE use yielded different results regarding their association with personal and environmental factors, as they were more influenced by factors related to physical activity and less associated with factors related to UE capacity.

**Conclusion:**

Healthcare providers should recognize the complexity of the relationship between UE use and impairment and consider additional factors when selecting assessments during rehabilitation to identify patients at risk of underutilizing their paretic arm in daily life. Future research in this domain should preconize relative UE use metrics or multi-sensors method to control for the effect of physical activity.

**Supplementary Information:**

The online version contains supplementary material available at 10.1186/s12984-025-01568-1.

## Introduction

Between 35% and 69% of individuals who experience a stroke will develop paresis in one of their upper extremities (UE) [1, 2]. These UE impairments will impede the individual’s ability to use their UE in daily activities, leading to a decline in their level of independence and quality of life [3, 4]. Rehabilitation after stroke can significantly improve UE capacity (i.e., the ability to execute tasks with the UE under controlled conditions). However, recent studies have shown that improved UE capacity during rehabilitation does not necessarily translate into increased use of the paretic UE in daily activities [5, 6]. Given that improving the daily use of the paretic UE is of greater importance than simply improving its capacity [7], it is essential to specifically assess UE use in everyday life and to develop a more comprehensive understanding of the factors that may influence it.

Upper extremity use can be assessed in multiple ways, either using questionnaires or wearable sensors [8]. When using self-reported questionnaires, like the Motor Activity Log (MAL), individuals often overestimate or underestimate their performance [9]. This discrepancies between self-reported and direct measures could be explained by the subjective nature of self-reported assessments and their susceptibility to recall and social-desirability bias [10, 11]. Moreover, using such questionnaires can be challenging in a population that frequently presents cognitive or language deficits [12, 13]. To overcome this challenge, the measure can be reported by a caregiver, but this has been shown to be less reliable and often impractical in hospital settings [14, 15]. Wrist-worn sensors, such as accelerometers, allow an ecological, objective, and valid measure of UE use in everyday life [8]. Accelerometers measure accelerations generated by arm movements and convert them into arbitrary units called activity counts over a predefined time epoch (generally 1 s). Accelerometers can quantify UE use using either intensity or duration metrics. Intensity metrics represent the total activity counts across all epochs, where duration metrics represent the sum of all epochs during which the UE was moving, using a minimum activity count threshold to determine the presence of movement during this period [16, 17]. As most studies use accelerometers on both wrists, accelerometry metric can also represent the use of the paretic UE alone, or the relative use of the paretic UE vs. the non-paretic UE (e.g., using a ratio). Thus, a large variety of accelerometry metrics have been used in the literature in order to quantify UE use in the stroke population, and each metric represents different aspects of UE use [18].

Recent reviews on UE use in the stroke population have focused on reporting the different methodological approaches employed or the validity of accelerometers [19–22]. A review with a clinical scope is needed, given the growing body of research exploring the relationship between paretic UE use and a myriad of factors ranging from neuroimaging markers to environmental factors. While a recent review did attempt to summarize the factors influencing UE use, it did not account for the different types of accelerometry metrics [23]. This is important because the association between UE use and a given factor changes significantly depending on the accelerometry metric that is used [24–26]. Some metrics have also demonstrated a better validity: for instance, the use ratio (duration of use of the paretic UE divided by duration of use of the non-paretic UE) shows better associations with UE impairment and capacity and is less influenced by the overall level of physical activity compared to unilateral metrics [17, 26, 27]. Finally, since many studies report multiple metrics simultaneously, it is crucial to extract each metric individually to draw accurate conclusions.

The first aim of this scoping review is to identify factors that are associated with UE use measured by accelerometry in the stroke survivors’ population. The second aim is to examine how these associations are influenced by the type of accelerometry metrics employed. This will guide future research by highlighting factors needing further investigation as well as informing methodological decisions regarding accelerometry metrics. It will also support clinicians by summarizing important considerations for paretic UE use in rehabilitation.

## Method

This scoping review followed the Preferred Reporting Items for Systematic reviews and Meta Analyses extension for Scoping Reviews (PRISMA-ScR) guidelines [28].

### Research strategy

Five databases were consulted: CINHAL (EBSCO), Embase (ELSEVIER), MEDLINE (EBSCO), Compendex (Engineering Village), and Web of Science Core Collection (CLARIVATE). The research strategy was based on three main concepts: (1) stroke; (2) accelerometers; (3) upper extremity. The keywords derived from those main concepts and the thesauri adapted for each database were used. The specific search strategy used for each database can be found in Supplementary Material (Table S1). The initial search in each database was launched on February 08, 2023, and updated on March 21, 2024. Articles were imported to EndNote (Clarivate Analytics, Philadelphia, PA) and then transferred to the Covidence online software (https://www.covidence.org), which was used to remove duplicates.

Article selection was made following these inclusion criteria: (1) included individuals with stroke; (2) used accelerometers to quantify the amount of UE use; (3) assessed the association between UE use and any factor related to the person or his environment (or tested the difference between two groups in the case of dichotomous variables, e.g. influence of sex or hand dominance)); (4) assessed all associated variables at a given point in time (i.e., transversal association); (5) reported univariate associations; (6) had a sample size ≥ 10; (7) were published through a peer-reviewed process; and (8) full text was available in English or French. Studies only assessing the relationship between two measures of UE use were not included. Therefore, association between UE use with accelerometry and the MAL, video annotation, or behavioral mapping were not included. The selection process was carried out independently by two of the authors using Covidence (L.G. and I.P.). A first screening was made based on titles and abstracts, and a final selection was made based on the articles’ full text. Any disagreement was resolved by a third person (C.M.).

### Data extraction

Data extraction for the selected articles was conducted by a single author (L.G). Variables extracted were (1) study design (2), aims (3), sample size (4), level of UE impairment (5), recovery stage and time since stroke (recovery stage followed SRRR guidelines [29]) (6), accelerometer method (model used, number of axes, sampling frequency, epoch length, time of wear, accelerometer metrics); (7) personal and environmental factors studied (classified according to the ICF model), and (8) associations between the factors and accelerometer’s metrics. The results reported were correlations (Pearson and Spearman), univariate regression, machine learning predictive model or between-group differences in the case of dichotomous variables (i.e., gender, dominance). When both univariate and multivariate analyses were presented in the same article, only univariates analyses were extracted. When results were available as part of a clinical trial, only baseline associations were extracted. When raw results were available, but associations were not analysed or presented in the articles, correlation and between-group analyses were performed using IBM SPSS Statistics (IBM SPSS Statistics 29, IBM Corp., NY, USA). Pearson’s correlation and t-test were used when distributions were normal, Spearman’s correlation and Mann-Whitney U test were used when the normality assumption was not met.

Only associations with accelerometry metrics quantifying UE use were extracted. Metrics related to movement quality (i.e., jerk) or global physical activity were not extracted. Accelerometry data collected only during therapy were not extracted, as paretic UE use would be heavily influenced by this context and would not represent UE use in everyday life.

### Data synthesis

The following benchmarks were used to determine the strength of the associations: perfect (*r* = 1.00), strong (*r* = 0.70–0.99), moderate (0.40–0.69), low (0.10–0.39), and no association (< 0.10) [30]. When an R^2^ from a regression was presented, the square root was applied to the result to compare the association with the same benchmarks. For differences between groups, as well as for the other types of analysis (i.e., machine learning predictive model), the presence or absence of an association was determined, but the strength of the association was not determined. When multiple associations were available for the same variable in a given study (i.e., results presented for different subgroups, different time points or multiple factors assessing the same construct), the average was selected for the synthesis. For example, if a study carried out three measurements over time and obtained two moderate associations and one strong association, a moderate association would be retained for the synthesis.

The International Classification of Functioning, Disability and Health (ICF) framework of the World Health Organization was used to classify the multiples factors that could potentially influence UE use. The ICF is a framework that provides a standardised way to describe and classify factors related to health and disability [31]. In order to reach a conclusion about the consistency of the association for a given variable across studies, the method described by Streber et al. was used [32]. Table 1 presents the summary method employed.

**Table 1:** Summary method

Summary code	% of studies supporting an association	Number of studies investigating the variable
0 = no association	0–33%	< 4
00 = no association	0–33%	≥ 4
? = inconsistent association	34–59%	< 4
?? = inconsistent association	34–59%	≥ 4
+ = consistent association	60–100%	< 4
++ = consistent association	60–100%	≥ 4

*Table adapted from Abid et al. [33]

Considering the great variability in the UE use metrics reported in the literature, they were divided into categories. First, it was determined whether the metric represented the utilization of the paretic UE alone (e.g., duration of use of the paretic MS) or the relative use of the paretic UE vs. the non-paretic UE. Examples of common relative UE use metrics are the use ratio, where duration of use of the paretic UE is divided by duration of use of the non-paretic UE, or the laterality index, where activity of the paretic UE is subtracted from the activity of the non-paretic UE and then normalized by the total activity of both UE [17, 34]. Definitions and equations of common UE use metrics can be found in Supplementary Material (Table S2). Then, the metric was classified as representing the duration of use (i.e., number of hours of daily use) or the intensity of use (i.e., number of activity counts or vector magnitude). This led to the following four categories: [1] absolute paretic UE use duration; [2] absolute paretic UE use intensity; [3] relative UE use duration; [4] relative UE use intensity.

## Results

**Fig. 1:**
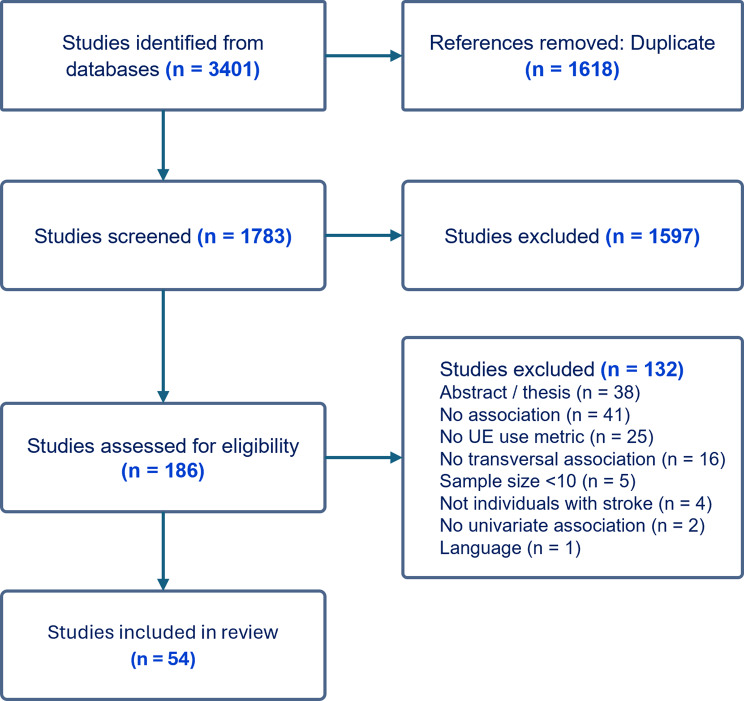
PRISMA flow diagram

The database search identified 3401 studies. Of these, 1499 were duplicates identified by Covidence and 119 more duplicates were identified manually. After titles and abstract screening, 1597 studies were classified as irrelevant. The remaining 186 studies were screened based on full text, and 54 articles were finally included in the review. The PRISMA flowchart is presented in Fig. 1.

### Studies characteristics

The characteristics of the studies included in the review are presented in Table 2. The time post-stroke ranged from acute to chronic stroke and arm impairment ranged from mild to severe deficits. Most studies collected accelerometry data in the community or in a stroke unit, and a few studies collected data in a laboratory setting (i.e., performing activities of daily living in a simulated living environment). Most of the studies came from North American (44%), European (33%), and Asian countries (15%).

**Table 2:** Studies characteristics

Firstauthor (year)	*N*	Time post stroke	Setting	UE use metric	Factors studied
Almubark (2018)	45	Chronic	Community	**Relative**: I	**Body S&F**: UE Kinematics, Compensatory movements, UE motricity **Activity**: Unimanual capacity
AltMurphy (2019)	28	Subacute	Stroke unit	**Relative**: I **Absolute**: I	**Personal**: Concordance **Environmental**: Weekend vs. Weekday
Andersson (2021)	26	Subacute	Stroke unit	**Relative**: I **Absolute**: I	**Health condition**: Stroke type, Time since stroke **Body S&F**: UE motricity, UE spasticity, UE pain, UE sensory, LE motricity, LE non-motor **Activity**: Mobility, General Autonomy **Personal**: Sexe, Age, Concordance
Bailey (2015)	46	Chronic	Community	**Relative**: T **Absolute**: T	**Health condition**: # comorbidities, time since stroke, # of stroke **Body S&F**: Cognition, Depression **Activity**: Unimanual capacity, General autonomy **Participation**: Physical activity **Personal**: Age, Concordance **Environmental**: Living arrangement
Bailey (2015)	48	Chronic	Community	**Relative**: I	**Activity**: Unimanual capacity **Personal**: Concordance
Barth (2020)	25	Acute	Stroke unit	**Relative**: T **Absolute**: T	**Body S&F**: UE motricity **Activity**: Unimanual capacity
Barth (2020)	78	Chronic	Community and laboratory setting	**Relative**: I, T **Absolute**: I	**Body S&F**: Compensatory movements
Bezuidenhout (2022)	40	Chronic	Community and laboratory setting	**Relative**: I	**Body S&F**: UE motricity **Activity**: Bimanual capacity **Personal**: Concordance
Bhatnagar (2020)	21	Chronic	Community	**Relative**: I, T **Absolute**: I, T	**Body S&F**: UE motricity **Activity**: Unimanual capacity
Bochniewicz (2017)	10	Chronic	Laboratory setting	**Absolute**: T^c^	**Activity**: Unimanual capacity
Chen (2023)	30	Chronic	Community	**Absolute**: T^a^	**Body S&F**: UE motricity
Chin (2021)	60	Subacute	Stroke unit	**Absolute**: T	**Health condition**: Stroke severity, Time post-stroke **Body S&F**: UE motricity, UE sensory, UE spasticity, UE pain, Balance, Cognition **Activity**: General autonomy, Fall risk **Participation**: Physical activity **Personal**: Age, Sex, Knowledge on UE, Concordance, Self-efficacy **Environmental**: Social support, Time spent in rehabilitation
Chin (2020)	60	Subacute	Stroke unit	**Relative**: I, T **Absolute**: T	**Body S&F**: UE motricity **Environmental**: Therapy **Personal**: Concordance
Demers (2024)	30	Chronic	Community	**Relative**: T	**Body S&F**: UE motricity **Activity**: Bimanual capacity **Personal**: Self-efficacy
DeNiet (2007)	17	Subacute and chronic	Community or Stroke unit	**Relative**: I **Absolute**: I	**Body S&F**: UE motricity
Doman (2016)	13	Subacute and chronic	Community (Outpatient)	**Relative**: I, T	**Health condition**: Time since stroke **Activity**: Unimanual capacity **Personal**: Age, Sex, Concordance
Duff (2022)	20	Chronic	Laboratory setting	**Relative**: I	**Body S&F**: UE motricity **Activity**: Bimanual capacity
Dusfour (2023)	19	Chronic	Community	**Relative**: T	**Body S&F**: UE motricity
Narai (2016)	19	Acute to subacute	Stroke unit	**Relative**: I **Absolute**: I	**Health condition**: Stroke severity **Body S&F**: UE motricity, LE motricity **Activity**: Unimanual capacity, General autonomy **Participation**: Physical activity
Flury (2021)	15	Chronic	Community (Outpatient)	**Relative**: T^b^ **Absolute**: T^b^	**Health condition**: Stroke severity **Body S&F**: UE motricity, Balance **Activity**: Unimanual capacity, General autonomy, Mobility **Participation**: Physical activity
Gebruers (2013)	129	Acute	Stroke unit	**Relative**: I **Absolute**: I	**Health condition**: Stroke severity **Body S&F**: UE motricity **Personal**: Concordance
Gebruers (2011)	130	Acute	Stroke unit	**Relative**: I **Absolute**: I	**Health condition**: Stroke severity **Body S&F**: UE motricity, UE oedema
Gebruers (2008)	39	Acute	Stroke unit	**Relative**: I **Absolute**: I	**Health condition**: Stroke severity **Body S&F**: UE motricity
Geed (2023)	31	Chronic	Laboratory setting	**Relative**: T^a^	**Body S&F**: UE motricity **Activity**: Unimanual capacity
Gulde (2024)	50	Subacute to chronic	Stroke unit	**Relative**: I, T	**Body S&F**: UE strength **Activity**: Unimanual capacity **Personal**: Concordance, Sex
Haaland (2012)	60	Chronic	Laboratory setting	**Absolute**: T^a^	**Personal**: Concordance
Hyakutake (2019)	10	Chronic	Community	**Relative**: I	**Health condition**: Time since stroke **Body S&F**: UE motricity **Activity**: Unimanual capacity **Personal**: Age, Gender, Concordance
Iacovelli (2019)	20	Acute	Stroke unit	**Relative**: I	**Health condition**: Stroke severity **Body S&F**: UE motricity **Personal**: Age, Gender, Concordance
Kokotilo (2010)	10	Chronic	Community	**Absolute**: I	**Health condition**: Ipsilesional fMRI biomarker, Contralesional fMRI biomarker
Lakhani (2017)	18	Chronic	Community	**Relative**: I	**Health condition**: Lesion volume, Time since stroke, myelination asymmetry between sensorimotor regions **Body S&F**: UE motricity **Activity**: Unimanual capacity **Personal**: Age, Concordance
Lang (2007)	34	Acute	Stroke unit	**Absolute**: T	**Body S&F**: UE motricity, UE pain, UE spasticity, UE sensory **Activity**: Unimanual capacity, General autonomy **Personal**: Concordance
Lee (2011)	16	Subacute	Stroke unit and community (multiple time points)	**Relative**: I **Absolute**: I	**Body S&F**: UE motricity
Lee (2020)	29	Chronic	Community	**Relative**: I	**Body S&F**: UE motricity **Activity**: Unimanual capacity **Personal**: Concordance
Leuenberger (2017)	10	Subacute and chronic	Community	**Relative**: I^b^, T^b^ **Absolute**: I^b^, T^b^	**Activity**: Unimanual capacity
Lum (2020)	10	Chronic	Laboratory setting	**Relative**: T	**Activity**: Unimanual capacity
Lundquist (2022)	87(T1) 67 (T2)	Subacute	Community	**Relative**: T	**Activity**: Unimanual capacity
Michielsen (2009)	17	Subacute and chronic	Stroke unit and community	**Relative**: I	**Body S&F**: UE motricity **Activity**: Unimanual capacity, Bimanual capacity
Otaki (2022)	25	Subacute	Stroke unit and Community (multiple time point)	**Relative**: I	**Body S&F**: Neglect, UE motricity **Activity**: Unimanual capacity
Rand (2015)	32	Chronic	Community	**Absolute**: I^b^	**Personal**: Age, Gender, Concordance
Reale (2023)	64	Acute	Stroke unit	**Relative**: I **Absolute**: I	**Health condition**: Stroke severity **Body S&F**: UE motricity
Reiterer (2008)	28	Acute and Subacute (Multiple time point)	Stroke unit and Community (multiple time point)	**Absolute**: I	**Health condition**: Stroke severity **Body S&F**: UE motricity **Activity**: General autonomy
Rinehart (2009)	29	Chronic	Laboratory setting	**Absolute**: T^a^	**Health condition**: Concordance
Shim (2014)	40	Chronic	Stroke unit	**Relative**: I **Absolute**: I	**Body S&F**: UE motricity
Thrane (2011)	31	Acute and subacute	Stroke unit (*n* = 23) or Community (*n* = 10)	**Relative**: T **Absolute**: T	**Body S&F**: UE motricity **Activity**: Mobility, General autonomy
Toba (2021)	35	Acute to chronic	Stroke unit or community	**Relative**: I	**Health condition**: Time since stroke **Body S&F**: UE motricity, Neglect, Somatosensory impairment, Visual field, Preferential gaze orientation, Anosognosia **Personal**: Sex, Age, Concordance, Education
Urbin (2014)	19	Chronic	Community	**Absolute**: T	**Health condition**: Homotopic rsFC, Heterotopic rsFC, Ipsilesional rsFC, Contralesional rsFC.
Urbin (2015)	27	Chronic	Community	**Relative**: I, T **Absolute**: I	**Activity**: Unimanual capacity
Uswatte (2006)	169	Subacute	Community	**Relative**: T **Absolute**: T	**Activity**: Mobility
VanderPas (2011)	45	Subacute and chronic	Community	**Relative**: I **Absolute**: I	**Body S&F**: Unimanual capacity, Mobility
Vier (2020)	31	Chronic	Community	**Relative**: T	**Activity**: Unimanual capacity
Waddell (2019)	29	Subacute	Stroke unit and Community	**Relative**: T	**Personal**: Self-efficacy
Wallich (2023)	60	Subacute	Community	**Relative**: T	**Activity**: Unimanual capacity
Wang (2011)	51	Chronic	Community	**Relative**: T	**Activity**: Bimanual capacity
Yamamoto (2023)	20	Subacute	Stroke unit	**Relative**: I **Absolute**: I	**Body S&F**: UE motricity **Activity**: Unimanual capacity, General autonomy

^a^ Unilateral movement, ^b^ Walking time removed, ^c^ Non-functional movements removed

*Concordance* concordance between the dominant UE and side of paresis, *fMRI* functional Magnetic Resonance Imaging, *I* Intensity, *LE* Lower Extremity, *rsFC* resting state functional connectivity, *S&F* Structure and Function, *T* time, *UE* Upper Extremity

A wide range of UE use metrics were used through the studies, often with multiple metrics used within a given study. The most reported metrics were relative UE use intensity metrics, where activity counts were cumulated over each epoch for both arms and then compared using a ratio or a delta count. Of the 54 included studies, 30 reported relative UE intensity metrics (56%), 21 reported relative UE duration metrics (39%), 19 reported absolute paretic UE intensity metrics (35%), and 15 reported absolute paretic UE duration metrics (28%). A posteriori decision was made to combine the results of the two categories of relative UE use metrics (relative intensity and relative duration) for the Results section, as the results obtained in both categories were similar. However, a comparison of results across the four different categories is available in Table S3 in the Supplementary Material. The result section will mostly focus on factors associated with relative UE use, as most of the studies presented relative UE use metrics. Also, it has been demonstrated that metrics of relative UE use have better validity than metrics of absolute UE use as they allow to control for the effect of physical activity (e.g., walking, whole-body-movement) [17, 27]. Differences in associations between the different metric categories will be presented at the end of the Results section.

A wide range of personal and environmental factors were also studied. Factors relative to body structures and functions were the most studied (37 studies, 69%), followed by activity limitations (32 studies, 59%), health condition (20 studies, 37%), and personal factors (18 studies, 33%). Only a few studies assessed factors relative to participation (5 studies, 9%) or environmental factors (4 studies, 7%).

### Factors associated with UE use

**Fig. 2:**
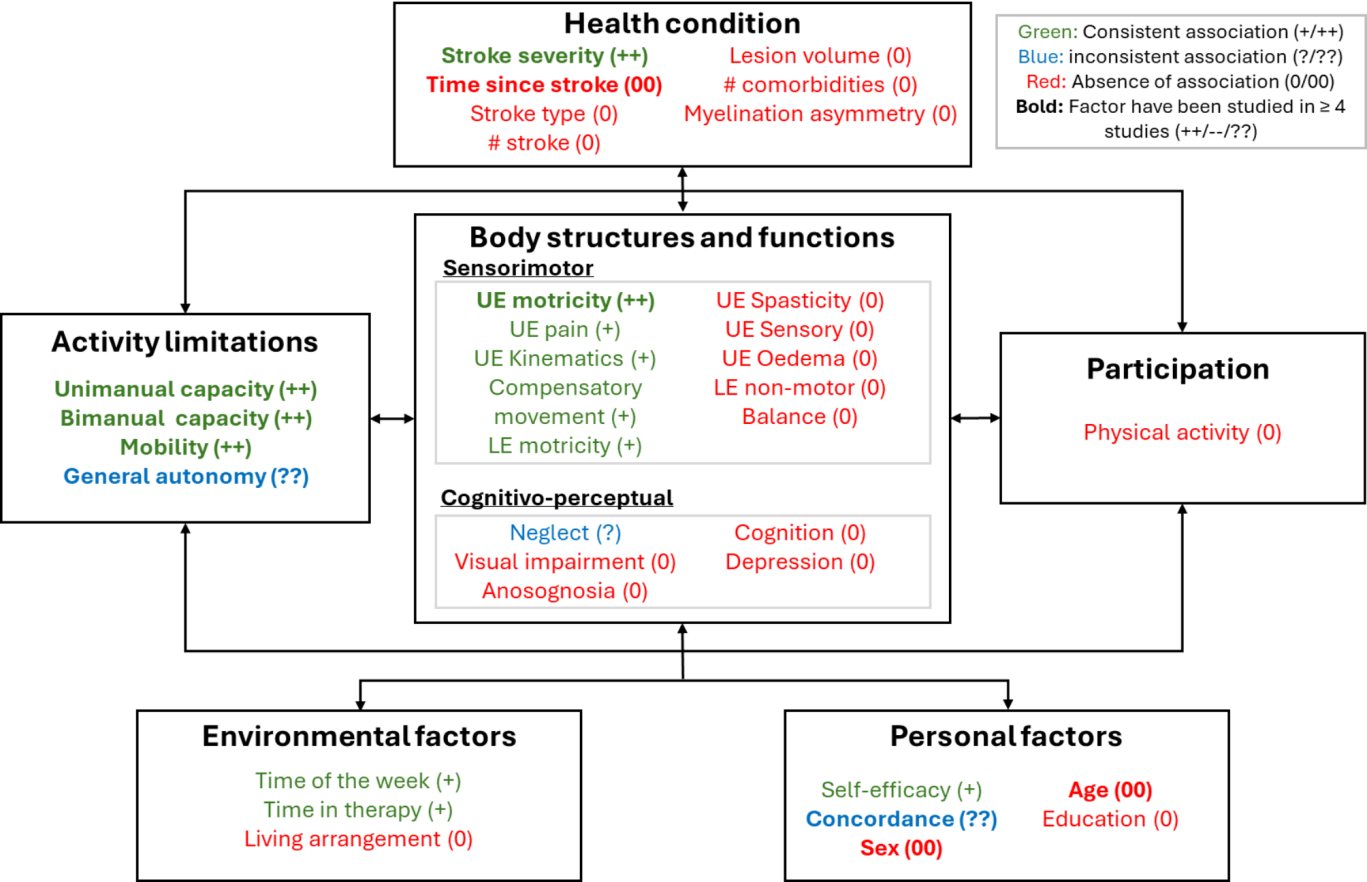
Classification of factors associated with relative UE use according to the ICF

Table 3 provides an overview of the factors associated with relative UE use. For each factor, it presents the number of studies that investigated it, the total number of participants across those studies, and the conclusions drawn regarding the presence of an association with relative UE use. A graphical summary based on the ICF framework is shown in Fig. 2.

**Table 3:** Summary of associations between personal and environmental factors and relative UE use

Factor studied	Assessment used	Stroke stage	# subjects	Relation	Conclusion
**Health condition**					
Stroke type		Subacute	26	1 No	0
Number of strokes		Chronic	46	1 No	0
Lesion volume		Chronic	18	1 No	0
Sensorimotor cortices myelination asymmetry	Sensorimotor cortex MWF	Chronic	18	1 No	0
Time since stroke		Acute to chronic	148	6 No	00
Stroke severity	NIHSS	Acute to subacute	401	6 Yes (M + S)	++
	NIHSS	Chronic	15	1 No	
Number of comorbidities		Chronic	46	1 No	0
**Body structures and functions**					
UE motricity	BRS Hand & UE, FMA-UE, NIHSS-UE, SAFE, MI, CMSA, Grip strength	Acute to chronic	982	26 Yes (M + S)	++
	FMA-UE	Chronic	59	3 No	
UE Spasticity	mAS	Subacute	26	1 No	0
UE Pain	FMA-UE Pain	Subacute	26	1 Yes (M)	+
UE Sensory	FMA-UE sensory, Hand tactile detection	Acute to chronic	61	2 No	0
UE Oedema		Acute	130	1 No	0
UE Kinematics	Motion capture system	Chronic	45	1 Yes	+
Compensatory movement	Trunk motion capture system, Video analysis	Chronic	123	2 Yes (M)	+
LE motricity	FMA-LE, BRS LE	Acute to subacute	45	2 Yes (M)	+
LE non-motor	FMA-LE sensory & pain, mAS	Subacute	26	1 No	0
Balance	Berg Balance Scale	Chronic	15	1 No	0
Neglect	CBS, GEREN, LeC, LiC, FT, C&R, Bisiach test	Acute to chronic	35	1 Yes (M)	?
	Dummy hand experimentation	Sub-acute	25	1 No	
Visual impairment	Visual field, preferred gaze orientation	Acute to chronic	35	1 No	0
Anosognosia	CBS	Acute to chronic	35	1 No	0
Cognition	Short blessed test	Chronic	46	1 No	0
Depression	CESDS	Chronic	46	1 No	0
**Activity limitations**					
Unimanual UE	ARAT, WMFT, SIS-Hand, STEF, BBT, TEMPA, NHPT	Acute to chronic	653	20 Y (S + M)	++
	ARAT, SIS-Hand	Chronic	49	3 No	
Bimanual UE	CAHAI, ABILHAND, Ad-AHA stroke	Sub-acute to chronic	158	5 Yes (S + M)	++
Mobility	10mWT, FAC, Independent walking 5STS, SIS-Mobility	Acute to subacute	226	3 Yes (M + L)	**++**
	TUG, 10mWT, SIS-Mobility	Subacute & chronic	60	2 No	
General autonomy	Independence in ADLs, Sunnaas ADL-Index, mRS	Acute to chronic	103	3 Yes (M)	??
	mRS, FIM	Acute to chronic	54	3 No	
**Participation**					
Physical activity	Number of steps, PAS	Acute to chronic	80	3 No	0
**Personal factors**					
Concordance		Acute to chronic	325	8 No	??
		Subacute to chronic	199	5 Yes	
Sex		Acute to chronic	154	6 No	00
Age		Acute to chronic	142	6 No	00
		Subacute	26	1 Yes (L)	
Education		Acute to chronic	35	1 No	0
Self-efficacy	CAHM, Self-perceived barriers	Subacute to chronic	59	2 Yes (M)	+
**Environmental factors**					
Time of the week	WE vs. WD	Sub-acute	28	1 Yes (M)	+
Living arrangement		Chronic	46	1 No	0
Time in therapy	OT, PT	Sub-acute	60	1 Yes	+

*+/++* consistent association in less or more than 4 study, *-/--* no association in less or more than 4 study, *?/??* inconsistent association in less or more than 4 study, *5STS* Five times Sit To Stand, *10mWT* Ten Meters Walking Test, *Ad-AHA* Adult Assisting Hand Assessment, *ARAT* Action Research Arm Test, *BBT* Box and Blocks Test, *BRS* Brunnstrom Recovery Stages, *CAHAI* Chedoke Arm and Hand Activity Inventory, *CAHM* Confidence in Arm and Hand Movement Scale, *CBS* Catherine Bergego Scale, *CESD* Center for Epidemiologic Studies Depression Scale, *CMSA* Chedoke-McMaster Stroke Assessment, *C&R* Comb and Razor test, *FAC* Functional Ambulation categorie, *FIM* Functional Independence Measure, *FMA* Fugl-Meyer Assessment, *FT* Fluff Test, *mAS* modified Ashworth Scale, *L* Low association, *LE* Lower Extremity, *LeC* Letter Cancellation test, *LiC* Line cancellation test, *M* Moderate association, *MI* Motricity Index, *MRI* Magnetic Resonance Imaging, *mRS* Modified Rankin Scale, *MWF* Myelin Water Fraction, *NHPT* Nine-Hole Peg Test,, *NIHSS* National Institutes of Health Stroke Scale, *OT* Occupational Therapy, *PAS* Physical Activity Scale, *PT* Physical Therapy, *S* Strong association *SAFE* Shoulder Abduction Finger Extension test, *SIS* Stroke Impact Scale, *STEF* Simple Test for Evaluating Hand Function, *TEMPA* Test d’Évaluation des Membres supérieurs des Personnes Âgées, *TUG* Timed Up and Go Test, *UE* Upper Extremity, *WD* Weekdays, *WE* Weekend, *WMFT* Wolf Motor Function Test

**Health condition.** The only factor influencing relative UE use related to health condition was stroke severity [26, 35–39] (6 strong to moderate associations, *n* = 401; 1 no association, *n* = 15). Stroke severity was assessed with the National Institutes of Health Stroke Scale (NIHSS) in all seven studies. No significant association was found for the following factors: stroke type [40], number of strokes [41], lesion volume [42], myelination asymmetry between sensorimotor regions [42], and number of comorbidities [41]. Time since stroke was not associated with relative UE use [40–46] (6 no association), however it is noteworthy that most studies assessing this factor had a sample composed of stroke survivors at the same stage recovery, and thus with low variability across subjects. Only one study did assess the effect of time with a sample ranging from acute to chronic stroke, but also failed to demonstrate an association with UE use [45]. The effect of time on UE use would be better assessed in longitudinal studies examining intra-individual changes over time, but this is beyond the scope of this review.

**Body structures and functions.** Upper extremity motor impairment was the most studied and the most consistently associated factor with relative UE use [24–26, 34–40, 42, 44–61] (26 strong to moderate associations, *n* = 982; 3 no association, *n* = 59). The UE motor impairment category, which encompasses a wide range of assessments representing several components of UE motricity (i.e., strength, active range of motion, ataxia, synergies, etc.), considers both proximal and distal UE motricity. Some studies have assessed both proximal and distal joint motricity or active range of motion and have shown that both were significantly associated with UE use [25, 34, 35]. In contrast, one study did find that elbow and wrist active range of motion (AROM), but not shoulder AROM were associated with UE use and that elbow flexion and grip strength were better associated with UE use than wrist and shoulder strength, highlighting the importance of distal motricity for functional use of the UE [62].

Four other factors related to body structures and functions were found to be significantly associated with relative UE use but have only been assessed in one or two studies: UE pain [40], UE kinematics quality [49], presence of compensatory movements [49, 63], and LE motricity [35, 40]. Conversely, seven other factors investigated in a single study showed no significant association with relative UE use: UE spasticity [40], sensory deficits [40], oedema [37], balance deficit [26], anosognosia [45], cognition [41], and depression [41]. Inconsistent association was observed with neglect [45, 54] (1 moderate association, *n* = 35; 1 no association, *n* = 25).

**Activity.** Unimanual capacity was consistently associated with relative UE use [6, 16, 24–26, 35, 41–44, 49, 54, 55, 57, 59–61, 64–69] (20 strong to moderate associations, *n* = 653; 3 no associations, *n* = 49). Bimanual capacity was less frequently studied, but also consistently associated with relative UE use [47, 50, 55, 56, 70] (5 strong to moderate associations, *n* = 158). Unimanual capacity was mostly assessed with the Action Research Arm Test (ARAT). Bimanual capacity was assessed with the Chedoke Arm and Hand Activity Inventory (CAHAI), ABILHAND and the Adult Assisting Hand Assessment (Ad-AHA). Mobility was also consistently associated with UE relative use [8, 26, 40, 48, 65] (3 moderate to low association, *n* = 226; 2 no association, *n* = 60), although to a lower extent. Mobility barely reached the threshold to be considered as consistently associated (i.e., 60%) and significant associations were only moderate to low in strength, whereas associations with unimanual and bimanual capacity were substantially more consistent (i.e., 87% and 100%, respectively) with stronger associations. Mobility assessments encompass measures related to speed (e.g., ten meter walk test, five times sit to stand test) and independence (e.g., Functional Ambulation Categories) in different activities related to mobility. An inconsistent association was observed with measures of general autonomy [26, 35, 40, 41, 48, 60] (3 moderate associations, *n* = 103; 3 no associations, *n* = 54).

**Participation.** Physical activity was not found to be associated with relative UE use [26, 35, 41] (3 no associations, *n* = 80). Physical activity was assessed with the Physical Activity Scale [41] or the daily number of steps assessed with lower extremity accelerometers [26, 35].

**Personal factors.** Self-efficacy was the only personal factor associated with relative UE use [47, 71]. The concordance between the dominant UE and side of paresis was inconsistently associated with relative UE use [16, 36, 39–45, 50, 51, 59, 61] (8 no associations, *n* = 325; 5 associations, *n* = 259). Many studies assess the effect of age [39–45] (6 no association, *n* = 142; 1 low association, *n* = 26) and sex [39, 40, 43–45, 61] (6 no association, *n* = 154) on UE use, but no associations with relative UE use were observed. The level of education was also not associated with relative UE use [45].

**Environmental factors.** Only a few studies assessed the influence of environmental factors on UE use. One study did report a significant effect of the time of the week, with higher use during weekdays then weekend [72]. Another study observed a higher relative UE use during rehabilitation therapy compared to the rest of the day [51]. Living arrangements (i.e., living with others versus alone) was not significantly associated with relative UE use [41].

### Differences in associations between duration and intensity of relative UE use

Although the relative usage metrics were highly comparable, a few discrepancies were identified. In regard to the association with stroke severity, a consistent association was present with intensity of relative UE use [35–39] (6 strong to moderate associations), yet no association was observed with duration of relative UE use [26] (1 no association). This difference might be explained by the lack of studies assessing this factor with relative duration metrics. Differences in associations with mobility and general autonomy were also observed, where relative duration metrics were more consistently associated with both factors. Association with concordance between the dominant UE and side of paresis also differed, with an absence of association with intensity of relative use [16, 36, 39, 40, 42–45, 50, 51, 59, 61] (4 associations and 8 no associations), but an inconsistent association with duration of relative use [41, 43, 51, 61] (2 associations and 2 no associations).

### Differences in associations between relative UE use and absolute paretic UE use

Associations with absolute paretic UE use duration were largely different from the associations with other use metrics. When compared with the relative UE use metrics, absolute paretic UE use duration was less associated with some factors related to the UE (inconsistent association with unimanual capacity and UE pain, no association with concordance between the dominant UE and side of paresis) and it was more often associated with factors not related to the UE (inconsistent association with balance, cognition, and physical activity; consistent association with general autonomy [26, 41, 62, 73]). However, while mobility was not associated with absolute paretic UE use duration [8, 26, 48], it was consistently associated with the other three metrics of UE use [8, 26, 40, 48, 65].

Although to a lesser extent, some differences were also observed with absolute paretic UE use intensity. As observed with the duration metric, absolute paretic UE use intensity was more consistently associated with general autonomy [35, 40, 60, 74] and physical activity [35], but less consistently associated with concordance between the dominant UE and side of paresis [36, 40, 72, 75]. A consistent association was also observed with age [40, 75], which was not present with all the other UE use metrics.

Associations with absolute paretic UE use metrics were reported for new factors that were not assessed with relative UE use metrics, including knowledge about importance of UE recovery and exercises [73] and contralesional and homotopic functional magnetic resonance imaging biomarkers [76]. Social context and time spent in rehabilitation were also studied with this metric, yet no significant associations were identified [73].

Details about differences in associations between the different UE use metrics are showed in Table S3 in Supplementary Material.

## Discussion

The first aim of this scoping review was to identify factors that are associated with UE use measured by accelerometry in stroke survivors. Several studies consistently reported associations between relative UE use and stroke severity, UE motor impairment, unimanual capacity, bimanual capacity, and mobility. Despite being less investigated, other factors such as the presence of UE pain and compensatory movements, neglect, and level of self-efficacy should also be considered in clinical reasoning regarding UE use. In contrast, there was a consistent lack of association between relative UE use and time since stroke, sex, and age.

The second aim of this review was to examine how these associations were influenced by the type of accelerometry metrics employed. Metrics of relative intensity and duration yielded similar results, with few minor differences. However, important discrepancies in associations with personal and environmental factors were observed between metrics of relative use and absolute paretic use. In this section, we will first discuss the core links between UE impairment, capacity and use and their implications for UE recovery. We will then discuss the factors that still require further investigation, and finally, the impact of the choice of different types of accelerometry metrics.

### Upper extremity impairment, capacity and use

Upper extremity impairment and unimanual capacity were the most studied factors and both were consistently associated with UE use. Studies conducting multivariate analysis also consistently report UE impairment and capacity as the factor explaining most of the variance in UE use [40, 49, 77]. Given these findings, those factors should be the primary focus of rehabilitation to increase paretic UE use in everyday life. However, several longitudinal studies failed to observe an increase in UE use when a decrease in impairment occurred [5, 6, 75, 78]. Looking at the individual trajectories of a large cohort, we can see that an improvement in capacity is associated with an increase in use only for half of the individuals, while a third will see their use stagnate during rehabilitation [79]. One explanation could be that despite a strong association between impairment and use, the association may be non-linear. Several studies corroborate this explanation by demonstrating a plateau in UE use when impairment is severe to moderate, and that beyond a certain threshold of impairment, UE use begins to increase [55, 80–82]. Examples of thresholds identified in the literature are a Fugl-Meyer score > 45.3 [81] or 50.6 [82], or a Wolf Motor Function Test score > 3.44 [80]. Thus, an increase in capacity may not translate in an increase in UE use if these thresholds are not exceeded. A second explanation is that UE use reached a plateau early in the subacute phase and remain stable over time, even with subsequent improvements in capacity [83]. This is corroborated by longitudinal studies conducted early after stroke which observed an increase in both capacity and UE use [71, 83]. The absence of an increase in UE use observed in the studies mentioned earlier may be attributed to the fact that measurements started after one-month post-stroke, when UE use may already have stabilised. This underlines the importance of interventions that directly target UE use, rather than focusing solely on impairments, to prevent this plateau in UE use. Interventions such as wrist-worn devices providing feedback on UE use or constraint-induced movement therapy have shown potential to improve UE use during later stages of stroke rehabilitation [84, 85]. A third explanation, that may co-exist with the two previous ones, is that other factors than mere UE impairment and capacity are implicated and could explain why some individuals do not improve UE use after stroke.

### Factors needing further investigation

Associations between UE use and stroke severity, UE impairment or capacity have been extensively studied. However, our understanding of the myriads of other factors that influence UE use after stroke remains incomplete. Many variables, such as neurological biomarkers, UE pain, neglect, and environmental factors, have only been investigated in one or two studies while other factors, such as dominance, have yielded inconsistent results. There is still a need to clarify the role of the factors that have been little studied or have generated heterogeneous results.

One such area in need of further exploration is the role of neuroanatomical and neurophysiological biomarkers on UE use. While stroke severity at the level of observed symptoms (NIHSS) was consistently associated with UE use, the relationship between neurological biomarkers and UE use remains unclear. In this review, only one study investigated stroke severity with anatomical biomarkers and found no significant association [42]. However, a longitudinal study investigating predictors of UE use identified the integrity of the corticospinal tract as a significant predictor, even after controlling for UE impairment [86]. The absence of association in the aforementioned study could be explained by evidence showing an absence of association between lesion volume and UE impairment [87]. More specific indicators, such as the site of ischemic penumbra or the corticospinal tract integrity, have shown stronger correlations with UE impairment and should be further explored as potential biomarkers of UE use [88, 89].

Bimanual capacity is a relevant factor to consider when looking at UE use, as most everyday tasks require the coordinated use of both hands [16]. Moreover, individuals who have experienced a stroke seldom use their affected UE unimanually, opting instead for a bimanual approach (i.e., stabilisation with the paretic UE) [90]. It is thus conceivable that bimanual capabilities may offer a more accurate predictor of paretic UE use. This review identified a consistent association between bimanual capacity and relative use of the UE but provided no indication that bimanual capacity would be more closely associated than unimanual capacity. However, most articles reviewed assessed bimanual capacity using a questionnaire (ABILHAND), rather than an objective performance-based assessment [50, 55, 70], or only collected accelerometry data in a laboratory setting [56]. The only remaining study, which used CAHAI and compared it with use in everyday life, showed that they were highly correlated [47]. Further investigation of bimanual function to examine whether bimanual capacity is a more reliable indicator of everyday use than unimanual capacity is warranted.

The effect of concordance between the dominant UE and side of paresis on relative UE use has been studied extensively (*n* = 14), but inconsistent results have been obtained across studies. One of the main limitations when assessing the effect of concordance is the difficulty to separate the effects of the lesioned hemisphere from the effect of hand dominance. There is a rationale for both effects, as hand dominance has a protective effect on impairment [91], and as lesion to the right hemisphere stroke is linked to neglect, which can affect paretic UE use [45]. In this review, only one study controlled for the effect of the affected hemisphere. Using a sample consisting of half left-handed and right-handed participants, all with a right-hemisphere lesion, the study identified an effect of dominance on the relative UE use [59]. However, the observed effect in this study is counterintuitive and contrary to other findings in the literature [16, 40, 41, 61], as a protective effect on use was observed when the non-dominant UE was affected. A statistical issue related to sample size may also explain the inconsistency of the results, as the effect of concordance is assessed by looking at the difference between groups whereas most of other factors are assessed with correlation. Small sample sizes will have less statistical power to demonstrate an effect, while correlations tend to be overestimated due to higher variability. Further evidence is required from large-sample studies that control for either the effect of dominance or the lesioned hemisphere.

The inconsistent association between neglect and relative UE use identified in this review could be explained by the fact that neglect is a broad concept and that different types of assessment were used. The literature on post-stroke neglect suggests that personal and extra personal neglect are distinct subtypes, and that functional tests are more sensitive than pencil and paper tests [92–94]. In an in-depth study of neglect by Toba et al., functional tests and assessments of personal neglect (e.g., Catherine Bergego Scale, Comb & Razor test, Fluff test) were consistently associated with UE use, where pencil and paper assessments of extra personal neglect were inconsistently associated (e.g., no association for Bells test, Line bisection and Landscape drawing vs. association for Letter cancelation and Clock drawing) [45]. Therefore, functional assessments of personal neglect may be the most effective method to detect the potential influence of neglect on UE use. Considering that neglect is an impairment that can be reduced through rehabilitation [95], additional research should be carried out on the relationship between neglect and UE use.

Upper extremity pain after stroke is common, especially when impairment is present at the UE [96, 97]. One study did find an association between UE pain and use, although this association was no longer significant when controlling for UE impairment [40]. However, studies using wearable sensors to assess activity and movements kinematics in populations with musculoskeletal pain, but without paresis, demonstrate associations with pain intensity and fear of movement (kinesiophobia) [98]. Further studies on pain and UE use are needed and kinesiophobia assessment should be included to explore whether pain can lead to avoidance of UE use after stroke.

Environmental factors were the least studied level of the ICF. However, a temporal effect on UE use was observed during rehabilitation, with better UE use during therapy and weekdays. This reinforces the need to offer more occupational opportunities and an enriched care environment to facilitate better use of the paretic UE and promote recovery [99]. A limitation related to accelerometry assessment when taking interest in environmental factors is that it is decontextualized. However, momentary assessments using cellphones are increasingly used in accelerometry studies to allow a more holistic comprehension on how the UE is used. When using this approach, new associations can be observed, as an association between social support and UE use, that was not revealed by aggregated accelerometry assessments [82]. Further research on environmental factors is warranted, as they remain underexplored. Employing methods like momentary assessment is recommended when assessing associations with environmental factors, as it enables better contextualization of the results.

### Impacts of accelerometry metrics types

The second aim of this review was to examine how the associations between UE use and personal or environmental factors were influenced by the type of accelerometry metrics employed. The results of this review clearly demonstrated that different metrics yield different results regarding their association with these factors. This highlights some of the limitations of accelerometry for measuring UE use, most particularly when using metrics of absolute paretic UE use. When compared with the relative UE use metrics, absolute paretic UE use was less often associated with some factors related to the UE (unimanual capacity, UE pain, concordance) and was more often associated with factors related to global function (i.e., balance, cognition, physical activity and general autonomy). Those results are congruent with validation studies that have shown that absolute paretic UE use metrics are more likely to be influenced by the level of physical activity compared to relative use metrics [17, 27]. This can be explained by the fact that during activities that involve whole-body movement (e.g., walking, sit-to-stand), accelerations will be detected at the UE and will be classified as UE use. An increase in walking time while wearing the sensors could therefore falsely be interpreted as an improvement in the amount of UE use. The use of metrics of relative use allows to assess the symmetry of the UE use, without being influenced by the overall volume of activity at the UE. However, the constant association between mobility measures with relative UE use metrics demonstrated in this review shows that relative UE use metrics could also be influenced by walking and level of physical activity, but to a lesser extent compared to absolute UE metrics. This is demonstrated in a study showing that when removing the walking periods using a chest accelerometer, there is an absence of association between relative UE use and mobility assessment [26], supporting the hypothesis that walking time could explain the association between mobility and relative UE use. In this regard, future studies should preconize metrics that measure relative UE use, such as use ratio or magnitude ratio, or use a multi-sensor methodology to factorize the influence of walking. Studies only using metrics of absolute UE use without controlling walking should be interpreted cautiously because results may be influenced by factors as walking and level of physical activity.

### Clinical implications

This review offers several insights for neurorehabilitation after stroke. First, the multifactorial nature of UE use demonstrated in this review supports the relevance of using accelerometers in clinical settings, given that it is not possible to infer the level of UE use solely based on impairments. Furthermore, this review highlights that UE use differs when outside of therapy [51]. Consequently, it becomes exceedingly difficult for clinicians to accurately assess UE use based on observations and assessments during rehabilitation. Accelerometry could allow clinicians to identify patients at risk of underutilizing their UE in daily life by assessing discrepancies between capacity and actual use.

Second, the variety of personal and environmental factors associated with UE use suggests that factors other than UE impairments could be targeted in rehabilitation to facilitate UE use in everyday life. Future research should investigate factors such as bimanual capacity, neglect, self-efficacy, and environment as potential intervention targets to promote UE use.

### Study limitations

A wide variety of accelerometry metrics were reported through the studies selected for this review, making comparison and synthesis of the results challenging. Although a categorization of metrics has been carried out to allow a certain homogeneity in the grouped metrics, notable discrepancies exist in calculation methods. For example, some studies removed walking period using a chest accelerometer [26, 67, 75], or used a machine learning algorithm to discard non-functional movements [57, 100]. Other studies using absolute paretic UE use duration metrics only measured UE use when the paretic UE was used unilaterally, discarding time when bimanual movements were made [82, 101, 102]. Even within the same calculation method, differences in sampling frequency, epoch length or thresholding method have been observed from one study to another, when it has been shown that changes in those parameters influence the metric value [103, 104]. These differences in accelerometry methods may contribute to the observed inconsistencies in the results.

This review is also limited to cross-sectional associations. Therefore, longitudinal studies assessing long term predictors of UE use have not been included. It is also important to consider that the results presented in this review are from univariate analyses and that associations have not been corrected for UE impairment. Some of the identified associations might be attributable to a mediating effect from UE impairment. For example, UE and LE motor impairment are moderately correlated and show similar recovery profiles [105, 106]. Consequently, the associations between UE use and LE motricity could be fully or partially mediated by UE impairment. The same phenomenon could be observed with stroke severity, UE quality of kinematic, UE pain or mobility as those factors are all associated with UE impairment [96, 107, 108]. Although multivariate analyses were not presented in this review, they allow to see the direct influence of those factors on UE use after controlling for UE impairment. Factors as concordance [77], UE spasticity [40], mobility [40, 48], type of stroke [77], physical activity [109] and compensatory movements [49] also explained a small but significant proportion of variance in UE use when added to UE impairment in multivariate analysis. However, in some studies, factors that were significant in univariate analyses, like concordance [6, 35, 40], pain [6, 40], LE motricity, mobility or general autonomy [40], were no longer significant when entered in multivariate analyses with UE impairment. In this regard, the results of this study should be interpreted accordingly, and future studies should preconize multivariate analysis approaches, allowing correction for the level of impairment.

An important methodological limitation of this study is that data extraction was conducted by a single author. The absence of a second reviewer for cross-verification may have increased the risk of errors or bias in the extraction process.

## Conclusion

Upper extremity use after stroke is mainly influenced by stroke severity, UE impairment, unimanual and bimanual capacity. Other factors such as UE pain, neglect, self-efficacy and concordance need to be further investigated to better understand their impact on UE use. It is crucial for healthcare providers to recognize the complexity of the relationship between UE use and impairment, and that other factors can influence paretic UE use. Assessments during rehabilitation care should be selected accordingly to identify patients at risk of underutilizing their UE in daily life. It is recommended that future studies preconize accelerometry metrics of relative UE use and employ multilevel analysis to account for the potential mediating effect of UE impairment.

## Electronic supplementary material

Below is the link to the electronic supplementary material.

Supplementary Material 1

## Data Availability

No datasets were generated or analysed during the current study.
