# Supplementary material for: Factors associated with upper extremity use after stroke: a scoping review of accelerometry studies
Source: J Neuroeng Rehabil. 2025 Feb 24;22:33. doi: 10.1186/s12984-025-01568-1 (PMC11849390; doi:10.1186/s12984-025-01568-1)
Supplement: Supplementary file 1 — Supplementary Material 1 [file 12984_2025_1568_MOESM1_ESM.docx]

**Table S1. Search strategies for all databases**

| CINHAL (EBSCO) | |
| --- | --- |
| S1 | TI (Accelerometer* OR Accelerometry OR Actigraph* OR "Wearable device*" OR "Inertial measurement unit*" OR IMU OR "Activity count*" OR "Wearable sensor*" OR Actiwatch* OR "Ambulatory monitoring" OR "Activity Monitoring" OR "Performance in daily life" OR "Activity in daily life" OR usage) OR AB (Accelerometer* OR Accelerometry OR Actigraph* OR "Wearable device*" OR "Inertial measurement unit*" OR IMU OR "Activity count*" OR "Wearable sensor*" OR Actiwatch* OR "Ambulatory monitoring" OR "Activity Monitoring "OR "Performance in daily life" OR "Activity in daily life" OR usage) |
| S2 | (MH "Accelerometry+") |
| S3 | S1 OR S2 |
| S4 | TI ("Upper extremit*" OR "upper limb*" OR hand OR hands OR arm OR arms) OR AB ("Upper extremit*" OR "upper limb*" OR hand OR hands OR arm OR arms) |
| S5 | (MH "Upper Extremity+") |
| S6 | S4 OR S5 |
| S7 | TI (stroke* OR "cerebrovascular accident" OR cva OR hemiplegia OR hemipar* OR paresis OR paretic OR apoplex* OR "brain ischemia*" OR "brain infarction*") OR AB (stroke* OR "cerebrovascular accident" OR cva OR hemiplegia OR hemipar* OR paresis OR paretic OR apoplex* OR "brain ischemia*" OR "brain infarction*") |
| S8 | (MH "Stroke+") OR (MH "Intracranial Hemorrhage+") OR (MH "Intracranial Embolism and Thrombosis+") OR (MH "Hemiplegia") |
| S9 | S7 OR S8 |
| S10 | S3 AND S6 AND S9 |

| EMBASE (Elsevier) | |
| --- | --- |
| S1 | **stroke***:ab,ti OR **'cerebrovascular accident*'**:ab,ti OR **cva**:ab,ti OR **hemiplegia**:ab,ti OR **hemipar***:ab,ti OR **paresis**:ab,ti OR **paretic**:ab,ti OR **apoplex***:ab,ti OR **'brain ischemia*'**:ab,ti OR **'brain infarction***:ab,ti |
| S2 | **'brain ischemia'**/exp OR **'brain infarction'**/de OR **'brain hemorrhage'**/exp OR **'cerebrovascular accident'**/de OR **'ischemic stroke'**/exp OR **'lacunar stroke'**/exp |
| S3 | **#1**OR **#2** |
| S4 | **'upper extremit*'**:ab,ti OR **'upper limb*'**:ab,ti OR **hand**:ab,ti OR **hands**:ab,ti OR **arm**:ab,ti OR **arms**:ab,ti |
| S5 | **'arm'**/de OR **'upper limb'**/de OR **'hand'**/de |
| S6 | **#4**OR **#5** |
| S7 | **accelerometer***:ab,ti OR **accelerometry**:ab,ti OR **actigraph***:ab,ti OR **'wearable device*'**:ab,ti OR **'inertial measurement unit*'**:ab,ti OR **imu**:ab,ti OR **'activity count*'**:ab,ti OR **'wearable sensor*'**:ab,ti OR **actiwatch***:ab,ti OR **'ambulatory monitoring'**:ab,ti OR **'activity monitoring'**:ab,ti OR **'performance in daily life'**:ab,ti OR **'activity in daily life'**:ab,ti OR **usage**:ab,ti |
| S8 | **'accelerometry'**/exp OR **'actimetry'**/exp OR **'ambulatory monitoring'**/exp |
| S9 | **#7**OR **#8** |
| S10 | **#3**AND **#6**AND **#9** |

| MEDLINE (EBSCO) | |
| --- | --- |
| S1 | AB ( stroke* OR "cerebrovascular accident" OR cva OR hemiplegia OR hemipar* OR paresis OR paretic OR apoplex* OR "brain ischemia*" OR "brain infarction*" ) OR TI (stroke* OR "cerebrovascular accident" OR cva OR hemiplegia OR hemipar* OR paresis OR paretic OR apoplex* OR "brain ischemia*" OR "brain infarction*" ) |
| S2 | (MH "Stroke+") OR (MH "Intracranial Hemorrhage+") OR (MH "Intracranial Embolism and Thrombosis+") OR (MH "Hemiplegia") |
| S3 | S1 OR S2 |
| S4 | AB ( Accelerometer* OR Accelerometry OR Actigraph* OR "Wearable device*" OR "Inertial measurement unit*" ORIMU OR "Activity count*"OR "Wearable sensor*"OR Actiwatch* OR "Ambulatory monitoring" OR "Activity Monitoring" OR "Performance in daily life" OR "Activity in daily life" OR usage ) OR TI (Accelerometer* OR Accelerometry OR Actigraph* OR "Wearable device*" OR "Inertial measurement unit*" OR IMU OR "Activity count*"OR "Wearable sensor*"OR Actiwatch* OR "Ambulatory monitoring" OR "Activity Monitoring" OR "Performance in daily life" OR "Activity in daily life" OR usage ) |
| S5 | (MH "Monitoring, Ambulatory") OR (MH "Accelerometry+") |
| S6 | S4 OR S5 |
| S7 | AB ( "Upper extremit*" OR "upper limb*" OR hand OR hands OR arm OR arms ) OR TI ( "Upper extremit*" OR "upper limb*" OR hand OR hands OR arm OR arms ) |
| S8 | (MH "Upper Extremity+") |
| S9 | S7 OR S8 |
| S10 | S3 AND S6 AND S9 |

| Compendex (Engineering Village) | |
| --- | --- |
| S1 | (({Upper extremity} OR {Upper extremities} OR {upper limb} OR {upper limbs} OR hand OR hands OR arm OR arms) WN KY) |
| S2 | ((stroke* OR {cerebrovascular accident} OR cva OR hemiplegia OR hemipar* OR paresis OR paretic OR apoplex* OR {brain ischemia} OR {brain infarction} OR {brain infarctions}) WN KY) |
| S3 | (({Wearable sensors} WN CV) OR ({Accelerometers} WN CV)) |
| S4 | ((Accelerometer* OR Accelerometry OR Actigraph* OR {Wearable device} OR {Wearable devices} OR {Inertial measurement unit} OR {Inertial measurement units} OR IMU OR {Activity count} OR {Activity counts} OR {Wearable sensor} OR {Wearable sensors} OR Actiwatch* OR {Ambulatory monitoring} OR {Activity Monitoring} OR {Performance in daily life} OR {Activity in daily life} OR usage) WN ALL) |
| S5 | S3 OR S4 |
| S6 | S1 AND S2 AND S5 |

| Web of Science Core Collection (CLARIVATE) | |
| --- | --- |
| S1 | TS=("Upper extremit*" OR "upper limb*" OR hand OR hands OR arm OR arms) |
| S2 | TS=(Accelerometer* OR Accelerometry OR Actigraph* OR "Wearable device*" OR "Inertial measurement unit*" OR IMU OR "Activity count*" OR "Wearable sensor*" OR Actiwatch* OR "Ambulatory monitoring" OR "Activity Monitoring" OR "Performance in daily life" OR "Activity in daily life" OR usage) |
| S3 | TS=(stroke* OR "cerebrovascular accident" OR cva OR hemiplegia OR hemipar* OR paresis OR paretic OR apoplex* OR "brain ischemia*" OR "brain infarction*") |
| S4 | S1 AND S2 AND S3 |

**Table S2.** Definitions and equations of common UE use metrics

| Metric category | Definition |
| --- | --- |
| Absolute paretic UE use duration | **Duration of use:** Summation of all epochs duration where movements were detected at the paretic UE. Expressed in minutes or hours per day (41).  $\sum_{\boldsymbol{i=1}}^{\boldsymbol{N}} \boldsymbol{T}_{\boldsymbol{paretic movements, i}}$ |
| Absolute paretic UE use intensity | **Signal magnitude area:** Area under the curve of the acceleration, calculated as the L1 norm of the acceleration vector averaged over a fixed epoch. Expressed as a mean of SMA of all epochs in m/s^2^ (72).  $SMA=\frac{1}{T} \int\left\vert a_{x} \right\vert+ \left\vert a_{y} \right\vert+\left\vert a_{z} \right\vert dt$ |
|  | **Median paretic magnitude:** Median of all epochs VM at the paretic UE (64).  $VM=\sqrt{{a_{x}}^{2}+{a_{y}}^{2}+{a_{z}}^{2}}$ |
|  | **Total paretic activity count:** Summation of AC at the paretic UE for all epochs (74).  $\sum_{\boldsymbol{i=1}}^{\boldsymbol{N}} \boldsymbol{AC}_{\boldsymbol{paretic, i}}$ |
| Relative UE use duration | **Use ratio:** Duration of use of the paretic UE divided by duration of use of the non-paretic UE (17).  $UR=\frac{Paretic UE duration of use}{Non­paretic UE duration of use}$ |
| Relative UE use intensity | **Magnitude ratio:** Natural log of the VM of the paretic UE divided by the VM of the non-paretic UE for an epoch. Expressed as a mean of MR of all epochs (16).  $MR=ln\left( \frac{{\sqrt{{a_{x}}^{2}+{a_{y}}^{2}+{a_{z}}^{2}}}_{paretic}}{{\sqrt{{a_{x}}^{2}+{a_{y}}^{2}+{a_{z}}^{2}}}_{non­paretic}} \right)$ |
|  | **Activity count ratio:** Ratio of the summation of AC for all epochs of the paretic UE divided by the magnitude of the acceleration of the non-paretic UE (42).  $AC ratio=\frac{\Sigma{AC}_{paretic}}{\Sigma{AC}_{non­paretic}}$ |
|  | **Differential activity:** Subtracting the AC of the paretic UE from the AC of the non-paretic UE (35). $\Delta AC=\Sigma{AC}_{non­paretic}-\Sigma{AC}_{paretic}$ |
|  | **Laterality index:** Subtracting the total AC of the paretic UE from the total AC of the non-paretic UE, normalized by the total AC of both UE (34).  $LI=\frac{\Sigma{AC}_{non­paretic} - \Sigma{AC}_{paretic}}{\Sigma{AC}_{non­paretic}+ \Sigma{AC}_{paretic}}$ |

*a* Acceleration on a single axis (x, y or z), *AC* Activity Count, *LI* Laterality Index, *MR* Magnitude ratio, *N* Total number of epochs, *SMA* Signal magnitude area; *T* epoch duration; *UE* Upper Extremity, *UR* Use Ratio, *VM* Vector Magnitude.

**Table S3. Comparison of results across the four different UE use metric categories**

| Factor studied | Relative intensity | | | Relative duration | | Absolute intensity | | | | | Absolute duration | | |
| --- | --- | --- | --- | --- | --- | --- | --- | --- | --- | --- | --- | --- | --- |
|  | # associated studies/total# study | | Summary code | # associated studies/total# study | Summary code | # associated studies/total# study | | Summary code | | | # associated studies/total# study | | Summary code |
| **Health condition** |  |  | |  |  |  |  | | |  | |  | |
| Stroke type | 0/1 | 0 | |  |  | 0/1 | | | 0 | |  |  | |
| Number of strokes |  |  | | 0/1 | 0 |  | | |  | | 0/1 | 0 | |
| Lesion volume | 0/1 | 0 | |  |  |  | | |  | |  |  | |
| Myelination asymmetry | 0/1 | 0 | |  |  |  | | |  | |  |  | |
| Time since stroke | 0/5 | 00 | | 0/2 | 0 | 0/1 | | | 0 | | 0/2 | 0 | |
| Stroke severity | 6/6 | ++ | | 0/1 | 0 | 5/6 | | | ++ | | 1/2 | ? | |
| Number of comorbidities |  |  | | 0/1 | 0 |  | | |  | | 0/1 | 0 | |
| **Body structures and functions** |  |  | |  |  |  | | |  | |  |  | |
| Ipsilesional fMRI biomarker |  |  | |  |  | 0/1 | | | 0 | | 0/1 | 0 | |
| Contralesional fMRI biomarker |  |  | |  |  | 1/1 | | | + | | 0/1 | 0 | |
| Homotopic fMRI biomarker |  |  | |  |  |  | | |  | | 1/1 | + | |
| Heterotopic fMRI biomarker |  |  | |  |  |  | | |  | | 0/1 | 0 | |
| UE motricity | 20/23 | ++ | | 8/9 | ++ | 12/12 | | | ++ | | 5/8 | ++ | |
| UE Spasticity | 0/1 | 0 | |  |  | 0/1 | | | 0 | | 0/2 | 0 | |
| UE Pain | 1/1 | + | |  |  | 1/1 | | | + | | 1/2 | ? | |
| UE Sensory | 0/2 | 0 | |  |  | 0/1 | | | 0 | | 0/2 | 0 | |
| UE oedema | 0/1 | 0 | |  |  | 0/1 | | | 0 | |  |  | |
| UE Kinematics | 1/1 | + | |  |  |  | | |  | |  |  | |
| Compensatory movement | 2/2 | + | | 1/1 | + | 1/1 | | | + | |  |  | |
| LE motricity | 2/2 | + | |  |  | 2/2 | | | + | |  |  | |
| LE non-motor | 0/1 | 0 | |  |  |  | | |  | |  |  | |
| Balance |  |  | | 0/1 | 0 |  | | |  | | 1/2 | ? | |
| Neglect | 1/2 | ? | |  |  |  | | |  | |  |  | |
| Visual impairment | 0/1 | 0 | |  |  |  | | |  | |  |  | |
| Anosognosia | 0/1 | 0 | |  |  |  | | |  | |  |  | |
| Cognition |  |  | | 0/1 | 0 |  | | |  | | 1/2 | ? | |
| Depression |  |  | | 0/1 | 0 |  | | |  | | 0/1 | 0 | |
| **Activity limitations** |  |  | |  |  |  | | |  | |  |  | |
| Unimanual UE | 13/15 | ++ | | 11/13 | ++ | 5/6 | | | ++ | | 4/7 | ?? | |
| Bimanual UE | 3/3 | + | | 2/2 | + |  | | |  | |  |  | |
| Mobility | 1/2 | ? | | 2/3 | + | 2/2 | | | + | | 1/3 | 0 | |
| Fall risk |  |  | |  |  |  | | |  | | 0/1 | - | |
| General autonomy | 1/3 | 0 | | 2/3 | + | 3/4 | | | ++ | | 3/4 | ++ | |
| **Participation** |  |  | |  |  |  | | |  | |  |  | |
| Physical activity | 0/1 | 0 | | 0/2 | 0 | 1/1 | | | + | | 1/3 | ? | |
| **Personal factor** |  |  | |  |  |  | | |  | |  |  | |
| Concordance | 4/12 | 00 | | 2/4 | ?? | 1/4 | | | 00 | | 1/6 | 00 | |
| Sexe | 0/6 | 00 | | 0/2 | 0 | 0/2 | | | 0 | | 0/1 | 0 | |
| Age | 1/6 | 00 | | 0/2 | 0 | 2/2 | | | + | | 0/2 | 0 | |
| Self-efficacy |  |  | | 2/2 | + |  | | |  | | 1/1 | + | |
| UE knowledge |  |  | |  |  |  | | |  | | 1/1 | + | |
| Education | 0/1 | 0 | |  |  |  | | |  | |  |  | |
| **Environmental factor** |  |  | |  |  |  | | |  | |  |  | |
| WE / WD | 1/1 | + | |  |  | 1/1 | | | + | |  |  | |
| Living arrangement |  |  | | 0/1 | 0 |  | | |  | | 0/1 | 0 | |
| Social context |  |  | |  |  |  | | |  | | 0/1 | 0 | |
| Time in therapy | 1/1 | + | | 1/1 | + |  | | |  | | 1/1 | + | |
| Time spent in rehabilitation |  |  | |  |  |  | | |  | | 0/1 | 0 | |

*+/++* consistent association in less or more than 4 study, *-/--* no association in less or more than 4 study, *?/??* inconsistent association in less or more than 4 study, *fMRI* functional Magnetic Resonance Imaging, *LE* Lower Extremity, *UE* Upper extremity, *WE* Weekend, *WD* Weekday.
